# Reply to: Contemporary treatment of children with critical and near-fatal asthma

**DOI:** 10.5935/0103-507X.20160064

**Published:** 2016

**Authors:** Steven L Shein, Richard H Speicher, Alexandre T Rotta

**Affiliations:** Division of Pediatric Critical Care Medicine, UH Rainbow Babies & Children's Hospital, Case Western Reserve University - Cleveland, OH, United States

We thank Drs. Colleti Jr and Carvalho for their interest in our recent publication in *Revista Brasileira de Terapia Intensiva* .^(1)^ We agree that avoidance of mechanical ventilation (MV) is preferable in pediatric intensive care unit (PICU) patients with critical asthma, but primarily to avoid MV-associated morbidity, as MV-associated mortality is exceptionally rare in the current era. Newth recently reported a mortality rate of 4.3% for children with near-fatal asthma in United States PICUs, which is lower than the 9.4% mortality rate among adults hospitalized nearly 2 decades ago with near-fatal asthma that was reported in the paper cited by Colleti Jr and Carvalho.^(2,3)^ Moreover, 10 of the 11 children who died in the recent PICU study had suffered cardiac arrest prior to the PICU admission and neurologic injury was the cause of death in nearly all of them, not intractable pulmonary disease.^(2)^ Still, our practice is to avoid MV whenever possible in children with critical asthma.

High-flow nasal cannula (HFNC) has been associated with favorable outcomes in many patient groups, including premature neonates, young children with bronchiolitis, and adults with acute hypoxemic respiratory failure.^(4-6)^ HFNC primarily improves gas exchange by washing out dead space, and also causes positive pharyngeal pressures that may be transmitted somewhat to the distal airways and cause a low-level of positive end-expiratory pressure (PEEP). Positive airway pressure effects of HFNC are extremely dependent on patient size, cannula diameter, and HFNC flow rate. Pharyngeal pressures of 5 to 7cmH_2_O have been generated at 5 to 8L/minute in premature neonates weighing ~1 to 4kg, but flows of 50L/minute are needed to generate similar pressures in adult-sized patients.^(7,8)^ It is unclear how much of that pharyngeal pressure is actually transmitted to the alveoli, but it is thought to be clinically insignificant under usual flows and in the range of ~1cmH_2_O, thus too low to fully explain the observed clinical benefits.^(9)^ Furthermore, generation of single-level PEEP (as opposed to BiPAP) in asthma may worsen hyperinflation without assisting inspiratory work, leading some to suggest that HFNC should be avoided in asthma.^(10)^ While we do not believe that HFNC is contraindicated in asthma since dead space washout may be helpful and generation of PEEP is likely trivial at typical flow rates, there are insufficient data reporting use of HFNC in PICU patients with critical asthma for us to have included it in our review. Hopefully, now that devices are available that allow for concurrent use of HFNC and continuous nebulized albuterol without the introduction of unconditioned bias flow to the circuit (i.e., Aerogen nebulizer), literature describing its use in pediatric critical asthma will likely become available.

We thank Colleti Jr and Carvalho for commenting on the use of intravenous magnesium in patients with critical asthma. Several studies performed in the emergency department setting show associations between magnesium infusions and favorable outcomes, including the provocative paper by Irazuzta cited by Colleti Jr and Carvalho (published after submission of our manuscript).^(11)^ Our focus was not pediatric asthma care in the emergency department, but the treatment of critical and near-fatal asthma in the PICU. Unfortunately, there is insufficient evidence to fully support the routine use magnesium in this latter cohort. Colleti Jr and Carvalho noted that other authors have reported different dosing strategies than ours (25 - 40mg/kg), such as a bolus of 50 - 75mg/kg followed by an infusion of 40mg/kg/hour. In the one study of 19 subjects treated with that regimen cited by Colleti Jr and Carvalho, 3 subjects (15.8%) had side effects related to its use and no associations with clinical outcomes were reported.^(12)^ More research is indeed needed on the use of both magnesium and HFNC in children with critical asthma.

Steven L Shein, Richard H Speicher and Alexandre T Rotta

Division of Pediatric Critical Care Medicine, UH Rainbow Babies & Children's Hospital, Case Western Reserve University - Cleveland, OH, United States
